# Mitigating the Associations of Kidney Dysfunction With Blood Biomarkers of Alzheimer Disease by Using Phosphorylated Tau to Total Tau Ratios

**DOI:** 10.1001/jamaneurol.2023.0199

**Published:** 2023-03-29

**Authors:** Shorena Janelidze, Nicolas R. Barthélemy, Yingxin He, Randall J. Bateman, Oskar Hansson

**Affiliations:** 1 Clinical Memory Research Unit, Department of Clinical Sciences Malmö, Lund University, Lund, Sweden; 2 Department of Neurology, Washington University School of Medicine, St Louis, Missouri; 3 The Tracy Family SILQ Center, St Louis, Missouri; 4 Skåne University Hospital, Malmö, Sweden

## Abstract

**Importance:**

Chronic kidney disease (CKD) has been associated with increased plasma concentrations of phosphorylated tau (p-tau) 217 and p-tau181, which potentially decreases their usefulness in the diagnostic workup of Alzheimer disease (AD).

**Objective:**

To investigate associations of CKD with plasma ratios of p-tau217 and p-tau181 to the corresponding unphosphorylated peptides in AD.

**Design, Setting, and Participants:**

This cross-sectional study included patients with mild cognitive impairment (cohort 1; enrollment in 2000-2005) and replication in cohort 2 from the Swedish BioFINDER-2 study, including both cognitively unimpaired individuals and those with cognitive impairment (enrollment in 2017-2022). All participants were from 2 memory clinics in Sweden and had plasma tau assessments and CKD status established within 6 months of plasma collection.

**Exposures:**

P-tau217 and p-tau181, unphosphorylated peptides (Tau212-221 and Tau181-190), and the ratios (pT217/T217 and pT181/T181) as well as estimated glomerular filtration rate (eGFR) as an indicator of CKD.

**Main Outcomes and Measures:**

Associations between plasma-soluble p-tau and CKD.

**Results:**

A total of 141 participants from cohort 1 (mean [SD] age, 72.2 [7.7] years; 82 [58.2%] women) and 332 participants from cohort 2 (172 with cognitive impairment and 160 cognitively unimpaired individuals; mean [SD] age, 69.8 [9.4] years; 169 [50.9%] women) were included. Higher eGFR was associated with increased levels of plasma p-tau217, p-tau181, Tau212-221, and Tau181-190 in individuals with cognitive impairment (cohort 1: *R *range, −0.24 to −0.59; *P* < .004; cohort 2: *R *range, −0.18 to −0.53; *P* < .02) and cognitively unimpaired individuals (cohort 2: *R* range, −0.44 to −0.50; *P* < .001). However, eGFR did not correlate with the pT217/T217 ratio in patients with cognitive impairment (cohort 1: *R*, −0.11; *P* = .19; cohort 2: *R*, −0.02; *P* = .78), and the correlations with pT217/T217 ratio were significantly attenuated in cognitively unimpaired individuals (difference: *R*, −0.14 [95% CI, −0.22 to −0.007]; *P* = .001). For p-tau217 and pT217/T217, the mean fold increases in amyloid-β positive (Aβ+) compared with Aβ− groups ranged from 2.31 (95% CI, 1.86-2.77) to 4.61 (95% CI, 3.39-5.83) in participants with cognitive impairment and from 1.26 (95% CI, 0.98-1.55) to 1.27 (95% CI, 0.94-1.59) in cognitively unimpaired individuals and were clearly higher than the mean fold increases in those with CKD compared with those without CKD, ranging from 0.05 (95% CI, −0.28 to 0.38) to 0.72 (95% CI, 0.25-1.19) in participants with cognitive impairment and from 0.09 (95% CI, −0.08 to 0.26) to 0.36 (95% CI, 0.19-0.52) in cognitively unimpaired individuals.

**Conclusions and Relevance:**

In this study, CKD was associated with increased plasma levels of soluble tau, but for p-tau217 the associations were considerably lower than the association with Aβ positivity. Importantly, the ratios, and especially pT217/T217, were less associated with CKD than p-tau forms alone and therefore are likely to more accurately reflect AD-related pathological changes.

## Key Points

**Question:**

Is chronic kidney disease (CKD) associated with changes in plasma concentrations of phosphorylated tau biomarkers (ie, p-tau217 and p-tau181) and corresponding percent phosphorylated tau to unphosphorylated tau ratios (pT217/T217 and pT181/T181)?

**Findings:**

In this cross-sectional study including 473 participants from 2 independent cohorts, CKD was associated with increased plasma concentrations of p-tau217 and p-tau181. However, associations of CKD with the percent ratios and with pT217/T217 in particular were clearly attenuated.

**Meaning:**

To mitigate the associations of comorbidities like CKD with the performance of plasma AD biomarkers, certain biomarker ratios and specifically pT217/T217 should be considered for implementation in clinical practice and drug trials.

## Teaser

This cross-sectional study investigates associations of chronic kidney disease with plasma ratios of phosphorylated tau 217 and 181 to the corresponding unphosphorylated peptides in Alzheimer disease.

## Introduction

Plasma biomarkers of amyloid-β (Aβ) and tau pathologies and in particular different variants of phosphorylated tau (p-tau) have shown great promise for the diagnosis and prognosis of Alzheimer disease (AD).^1^ However, in a recent report from the population-based Mayo Clinic Study of Aging cohort consisting mainly of cognitively normal participants, several comorbidities were found to be associated with higher plasma concentrations of p-tau181 and p-tau217.^2^ The strongest associations were seen for chronic kidney disease (CKD) and thresholds for abnormality in the biomarker levels appeared to vary across subsamples including or excluding participants with CKD. These findings highlighted the potential need to take into account the presence of comorbidities when implementing plasma biomarkers in the clinical workup of AD or in drug trials in the future. However, one point to consider is that CKD most likely affects, nonspecifically, the total blood pool of tau and Aβ through reduced glomerular filtration rate. Consequently, the ratios of tau peptides might be normalized for the CKD-related changes in blood biomarker levels and as a result less affected. Further, we wanted to study the associations of CKD with plasma p-tau in patients with cognitive impairment because this is the patient population most likely to undergo AD biomarker testing in clinical practice. Therefore, we measured plasma concentrations of p-tau217 and p-tau181, as well as the corresponding unphosphorylated tau peptides, 212-221 (Tau212-221) and 181-190 (Tau181-190) in patients with mild cognitive impairment in a first cohort. We examined associations of CKD with individual tau biomarkers (p-tau217, p-tau181, Tau212-221, and Tau181-190) and importantly with the pT217/T217 and pT181/T181 ratios. We corroborated findings in the mild cognitive impairment cohort (cohort 1) in a second replication cohort (cohort 2), which included participants with cognitive impairment and cognitively unimpaired participants.

## Methods

Study participants were recruited at the Memory Clinic at Skåne University Hospital in Malmö, Sweden, between October 2000 and December 2005 in cohort 1 and between May 2017 and January 2022 in cohort 2. All participants in cohort 1 (eTables 1 and 2 in Supplement 1) had a clinical diagnosis of mild cognitive impairment.^3,4^ Cohort 2 (eTables 3 and 4 in Supplement 1) included a cognitively unimpaired group comprising cognitively healthy controls and individuals with subjective cognitive decline and another group of patients with cognitive impairment comprising those with mild cognitive impairment or dementia from the Swedish BioFINDER-2 study.^5^ All participants who had plasma tau assessments using immunoprecipitation mass spectrometry (IP-MS) and CKD status established within 6 months of plasma collection were included from both cohorts. Both studies were approved by the Regional Ethics Committee in Lund, Sweden. All participants provided written informed consent. This study followed the Strengthening the Reporting of Observational Studies in Epidemiology (STROBE) reporting guideline.

Plasma levels of p-tau217, p-tau181, Tau212-221, Tau181-190, and pT217/T217 and pT181/T181 ratios were measured using an in-house multiplex IP-MS as previously described.^6^ Plasma concentrations of Aβ40 and Aβ42 were determined with immunoassays (ADx Neurosciences) in cohort 1 and antibody-free liquid chromatography mass spectrometry (Araclon Biotech)^7^ in 212 participants from cohort 2. Cerebrospinal fluid (CSF) Aβ40 and Aβ42 concentrations were assessed using Meso Scale Discovery immunoassays in cohort 1 and using the Elecsys (Roche Diagnostics) or Lumipulse G (Fujirebio) immunoassays in cohort 2. All samples were analyzed by staff blinded to the clinical data. A total of 301 participants in cohort 2 had [^18^F]RO948 tau positron emission tomography (tau-PET) as previously described.^8^ [^18^F]RO948 standardized uptake value ratio was obtained for a temporal meta-ROI corresponding to Braak I-IV regions.^9^ Further details of cohort inclusion/exclusion criteria, sample collection, and analysis and tau-PET are described in the eMethods in Supplement 1. Study participants were classified as Aβ negative (Aβ−) or Aβ positive (Aβ+) based on the CSF Aβ42/40 ratio using previously described thresholds of 0.07 in cohort 1^4,10^ and 0.08 (Elecsys) or 0.072 (Lumipulse G) in cohort 2.^11,12^ Estimated glomerular filtration rate (eGFR) was used as an indicator of CKD. We used a commonly accepted Chronic Kidney Disease Epidemiology Collaboration (CKD-EPI) equation to calculate eGFR based on creatinine, age, sex, and race.^13^ We also performed a sensitivity analysis using the Lund-Malmö revised equation that has shown better performance than the CKD-EPI equation in a Swedish population.^14^ Participants were considered to have CKD based on the threshold of less than 60 mL/min/1.73m^2^, which is accepted as functional criteria for CKD and to differentiate normal/high eGFR from different stages of reduced eGFR.^15^

### Statistical Analysis

SPSS statistical software, version 28 (IBM) and R version 4.1.2 (NS RStudio) were used for statistical analysis. Correlations between eGFR and plasma biomarkers were examined with the Spearman test. The 95% CIs estimated from 2000 bootstrap iterations were used to test differences in the correlation coefficients. Group differences in the biomarker levels were assessed using univariate linear regression models with fold change in biomarker levels as outcome variables adjusting for the potential confounding association of age, sex, and CSF Aβ42/40 status. In cohort 2 where participants had tau-PET, we also included tau-PET–standardized uptake value ratio as a covariate in the regression models. Two-sided *P* < .05 was considered statistically significant.

## Results

### Original Cohort of Patients With Mild Cognitive Impairment (Cohort 1)

Cohort 1 included 141 participants with mild cognitive impairment with a mean (SD) age of 72.2 (7.7) years; 82 (58.2%) were women, and 67 (47.5%) had abnormal CSF Aβ42/40 (eTables 1 and 2 in Supplement 1).

We first tested correlations between eGFR and plasma p-tau biomarkers as shown in the Table and eFigure 1 in Supplement 1. Lower eGFR levels (indicative of kidney dysfunction) were associated with higher plasma concentrations of p-tau217, Tau212-221, p-tau181, and Tau181-190 (range: *R*, −0.24 [95% CI, −0.41 to −0.07] to −0.59 [95% CI, −0.70 to −0.47]; *P* < .004) with a significantly lower correlation coefficient seen for p-tau217 than p-tau181 (difference: *R*, 0.26 [95% CI, 0.16-0.36]; *P* < .001). Interestingly, we did not find significant correlation between eGFR and plasma pT217/T217 (*R*, −0.11 [95% CI, −0.28 to 0.06]; *P* = .19). Furthermore, the correlation coefficient for pT217/T217 was significantly lower compared with the correlation coefficients for p-tau217 (difference: *R*, −0.13 [95% CI, −0.19 to −0.08]; *P* < .001) and Tau212-221 (difference: *R*, −0.41 [95% CI, −0.60 to −0.23]; *P* < .001). Similarly, significantly lower correlation coefficients were seen for pT181/T181 than p-tau181 (difference: *R*, −0.28 [95% CI, −0.38 to −0.19]; *P* < .001) and Tau181-190 (difference: *R*, −0.38 [95% CI, −0.52 to −0.24]; *P* < .001). However, in contrast to pT217/T217, correlations between eGFR and pT181/T181 were statistically significant although weak (*R*, −0.22 [95% CI, −0.36 to −0.06]; *P* = .01).

**Table.** Spearman Correlations Between Plasma Tau Peptides and eGFR

Biomarker	*R* (95% CI)^a^	*P* value	Difference vs pT217/T217	
			*R* (95% CI)^a^	*P* value
**Cohort 1: mild cognitive impairment**				
p-tau217	−0.24 (−0.41 to −0.07)	.004	−0.13 (−0.19 to −0.08)	<.001
Tau212-221	−0.52 (−0.64 to −0.38)	<.001	−0.41 (−0.60 to −0.23)	<.001
pT217/T217	−0.11 (−0.28 to 0.06)	.19	NA	NA
**Cohort 2: cognitively unimpaired**				
p-tau217	−0.47 (−0.59 to −0.34)	<.001	−0.14 (−0.22 to −0.07)	.001
Tau212-221	−0.44 (−0.57 to −0.29)	<.001	−0.11 (−0.31 to 0.09)	.29
pT217/T217	−0.33 (−0.46 to −0.18)	<.001	NA	NA
**Cohort 2: cognitive impairment**				
p-tau217	−0.18 (−0.32 to −0.02)	.02	−0.16 (−0.21 to −0.11)	<.001
Tau212-221	−0.52 (−0.62 to −0.40)	<.001	−0.50 (−0.67 to −0.33)	<.001
pT217/T217	−0.02 (−0.17 to 0.13)	.78	NA	NA
**Biomarker**	***R* (95% CI)^a^**	***P* value**	**Difference vs pT181/T181**	
			***R* (95% CI)^a^**	***P* value**
**Cohort 1: mild cognitive impairment**				
p-tau181	−0.50 (−0.62 to −0.36)	<.001	−0.28 (−0.38 to −0.19)	<.001
Tau181-190	−0.59 (−0.70 to −0.47)	<.001	−0.38 (−0.52 to −0.24)	<.001
pT181/T181	−0.22 (−0.36 to −0.06)	.01	NA	NA
**Cohort 2: cognitively unimpaired**				
p-tau181	−0.48 (−0.60 to −0.34)	<.001	−0.31 (−0.43 to −0.19)	<.001
Tau181-190	−0.50 (−0.61 to −0.37)	<.001	−0.33 (−0.51 to −0.15)	<.001
pT181/T181	−0.17 (−0.32 to −0.01)	.03	NA	NA
**Cohort 2: cognitive impairment**				
p-tau181	−0.39 (−0.52 to −0.25)	<.001	−0.34 (−0.45 to −0.23)	<.001
Tau181-190	−0.53 (−0.65 to −0.41)	<.001	−0.48 (−0.64 to −0.32)	<.001
pT181/T181	−0.06 (−0.21 to 0.11)	.45	NA	NA

Abbreviations: eGFR, estimated glomerular filtration rate; NA, not applicable; p-tau, phosphorylated tau.

^a^ Data are shown as Spearman correlation coefficients (*R*).

**Figure 1.**
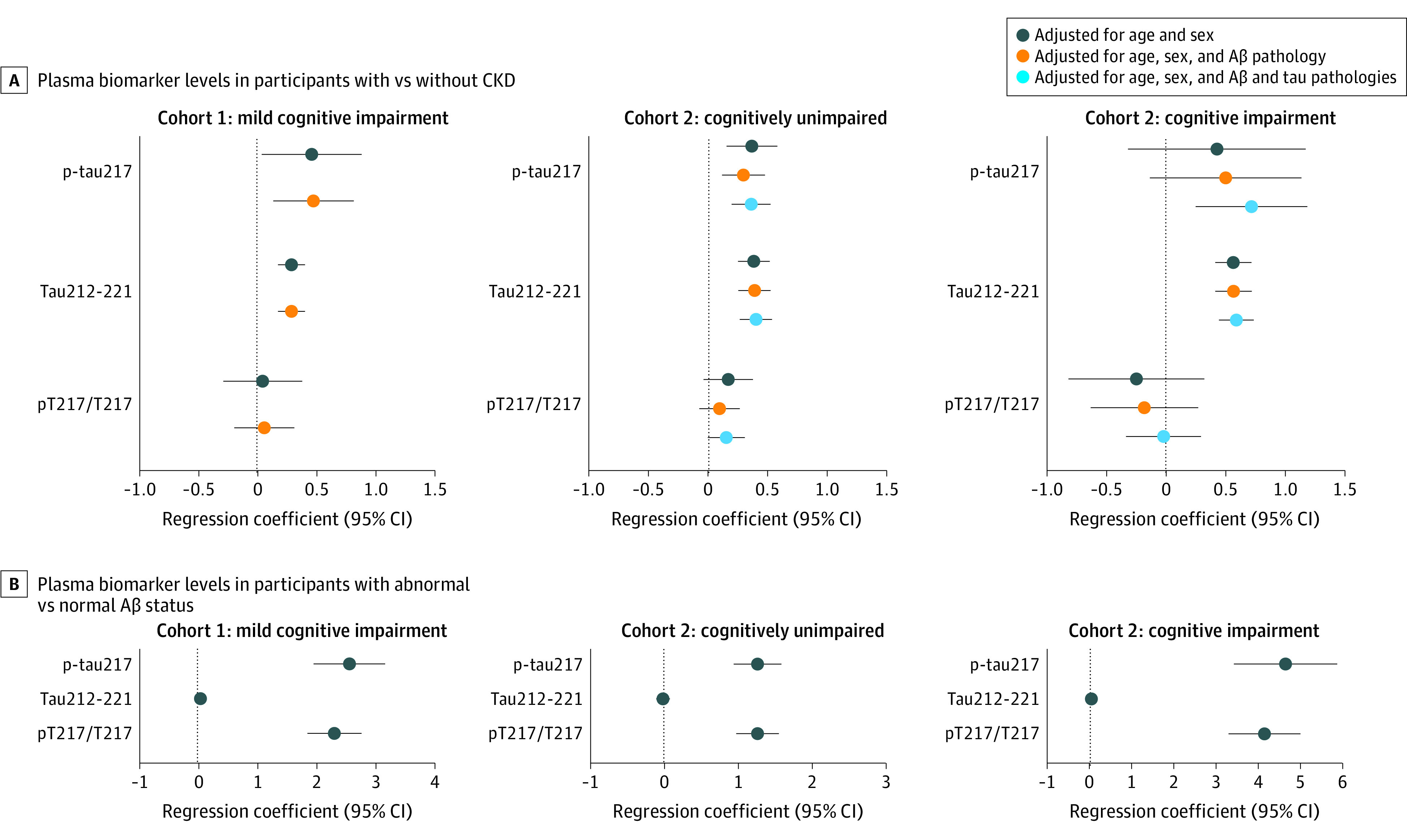
Associations of Plasma of Phosphorylated Tau (p-tau) 217, Tau212-221, and pT217/T217 With Chronic Kidney Disease (CKD) and Amyloid-β (Aβ) Status Fold increase in plasma biomarker levels in participants with CKD (CKD+) compared with those without CKD (CKD−) in cohort 1 (mild cognitive impairment, n = 141) and cohort 2 (cognitively unimpaired, n = 146 and cognitive impairment, n = 154) and in participants with abnormal compared with normal Aβ status in cohort 1 (mild cognitive impairment, n = 141) and cohort 2 (cognitively unimpaired, n = 146 and cognitive impairment, n = 154). Data are coefficients with 95% CI from linear regression models. Different x-axis scales in panels A and B reflect different magnitudes in fold changes for CKD and Aβ status. Aβ status was defined using cerebrospinal fluid Aβ42/40.

**Figure 2.**
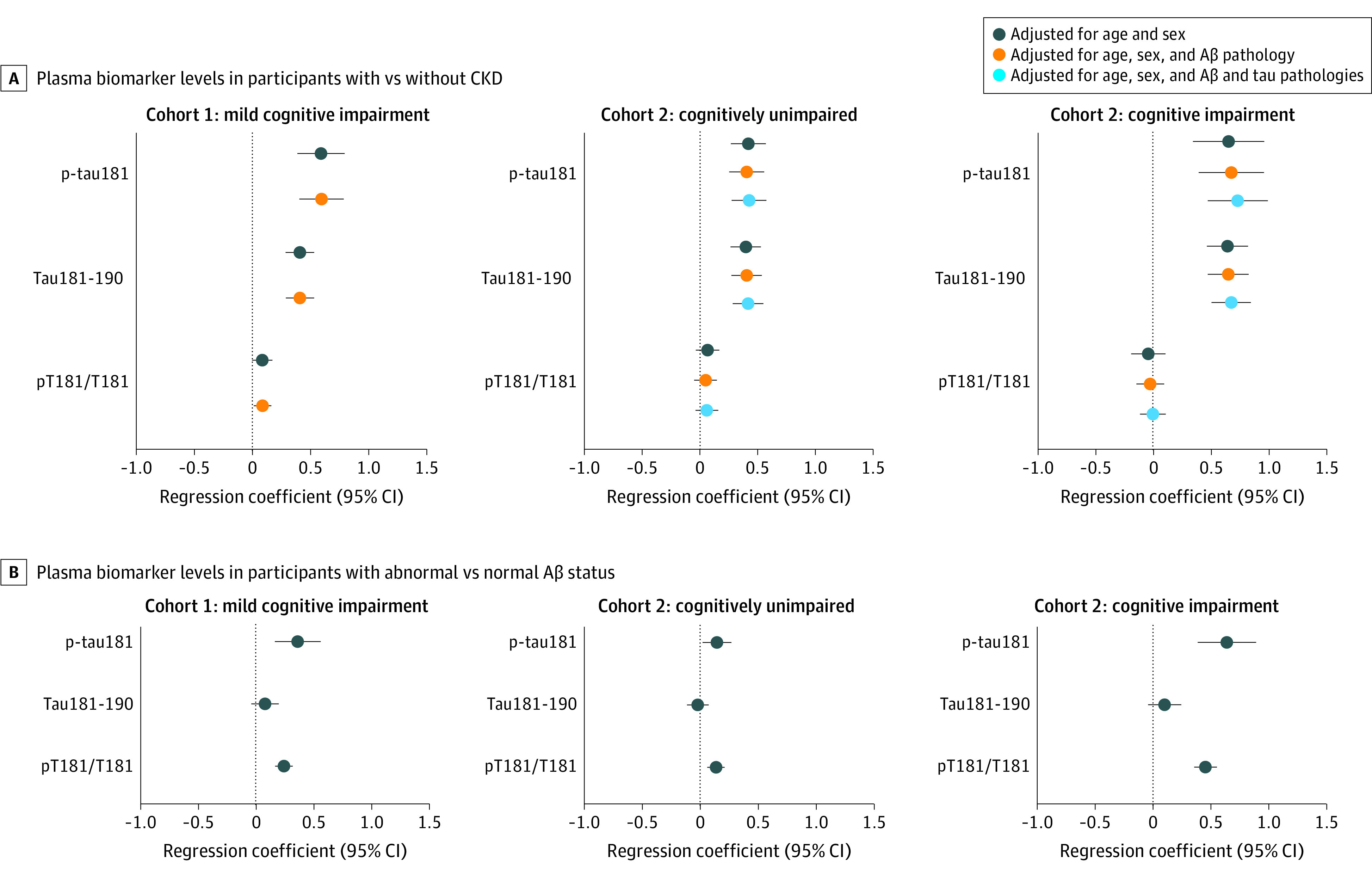
Associations of Plasma of Phosphorylated Tau (p-tau) 181, Tau181-190, and pT181/T181 With Chronic Kidney Disease (CKD) and Amyloid-β (Aβ) Status Fold increase in plasma biomarker levels in participants with CKD (CKD+) compared with those without CKD (CKD−) in cohort 1 (mild cognitive impairment, n = 141) and cohort 2 (cognitively unimpaired, n = 146 and cognitive impairment, n = 154) and in participants with abnormal compared with normal Aβ status in cohort 1 (mild cognitive impairment, n = 141) and cohort 2 (cognitively unimpaired, n = 146 and cognitive impairment, n = 154). Data are coefficients with 95% CI from linear regression models. Aβ status was defined using cerebrospinal fluid Aβ42/40.

We next compared the plasma p-tau levels between participants with CKD (CKD+) or without CKD (CKD−) (eTable 2 in Supplement 1). In line with the correlations results, plasma p-tau217, Tau212-221, p-tau181, and Tau181-190 were all increased in CKD+ compared with CKD− (fold increase range, 0.29 [95% CI, 0.18-0.41] to 0.60 [95% CI, 0.41-0.79]; *P* < .001) (Figure 1 and Figure 2). We also observed significantly higher pT181/T181 in CKD+ even though fold increase compared with CKD− was lower compared with p-tau181 and Tau181-190 (Figure 2A). Importantly, there were no significant differences between CKD+ and CKD− for pT217/T217 (fold increase, 0.05 [95% CI, −0.28 to 0.38] and 0.06 [95% CI, −0.19 to 0.32]; *P* > .63) (Figure 1A). Notably, for p-tau217 and pT217/T217, the fold increases in CKD+ vs CKD− (fold increase range, 0.05 [95% CI, −0.28 to 0.38]; *P* = .63 to 0.48 [95% CI, 0.14-0.82]; *P* = .006) were lower than the fold increases in Aβ+ compared with Aβ− (2.56 [95% CI, 1.96-3.17] and 2.31 [95% CI, 1.86-2.77]; *P* < .001) (Figure 1A and B). These results were verified when using the Lund-Malmö revised equation to calculate eGFR (eTable 5 and eFigure 2A in Supplement 1), except that there were no significant associations between CKD− status and pT181/T181 (eFigure 2A in Supplement 1). Finally, in line with already published data we found that eGFR was associated with plasma Aβ42 and Aβ40 but not with the Aβ42/40 ratio (eTable 6 and eFigure 3 in Supplement 1).^16,17^

### Validation Cohort of Cognitively Unimpaired Participants and Patients With Cognitive Impairment (Cohort 2)

The findings were replicated in cohort 2 (n = 332) including 160 cognitively unimpaired participants (104 cognitively healthy controls, 56 with subjective cognitive decline) and 172 patients with cognitive impairment (98 with mild cognitive impairment and 74 with dementia). The mean (SD) age in cohort 2 was 69.8 (9.4) years, 169 (50.9%) were women, and 172 (51.8%) had abnormal CSF Aβ42/40 (eTables 3 and 4 in Supplement 1). Correlations of eGFR with p-tau217, Tau212-221, p-tau181, and Tau181-190 were similar between the cognitive impairment groups in cohort 2 and cohort 1 (Table; eFigures 1 and 4 in Supplement 1). For p-tau217, the correlations were somewhat stronger in cognitively unimpaired participants (*R*, −0.47 [95% CI, −0.59 to −0.34]; *P* < .001) than patients with cognitive impairment (cohort 1: *R*, −0.24 [95% CI, −0.41 to −0.07]; *P* = .004; cohort 2: *R*, −0.18 [95% CI, −0.32 to −0.02]; *P* = .02) (Table; eFigures 4 and 5 in Supplement 1). In keeping with the results from cohort 1, correlations with pT217/T217 and pT181/T181 were significantly attenuated in both the cognitively unimpaired group and the cognitive impairment group from cohort 2, while nonsignificant in patients with cognitive impairment (difference range: *R*, −0.14 [95% CI, −0.22 to −0.07] to −0.50 [95% CI, −0.67 to −0.33]; *P *≤* *.001) (Table).

P-tau217, Tau212-221, p-tau181, and Tau181-190 were all increased in CKD+ compared with CKD− (fold increase range: 0.29 [95% CI, 0.11-0.48] to 0.73 [95% CI, 0.47-0.99]; *P* < .002), except in patients with cognitive impairment, there were no significant differences in p-tau217 in the models not including Aβ and tau pathologies as covariates (Figure 1 and Figure 2). Similar to findings in cohort 1, no differences were seen in pT217/T217 between CKD+ and CKD− in either cognitively unimpaired participants or patients with cognitive impairment (Figure 1). Furthermore, CKD was not associated with altered pT181/T181 (Figure 2). The fold changes in p-tau217 and pT217/T217 in CKD+ vs CKD− ranged from −0.02 (95% CI, −0.34 to 0.30) to 0.72 (95% CI, 0.25-1.19) in patients with cognitive impairment and from 0.09 (95% CI, −0.08 to 0.26) to 0.36 (95% CI, 0.19-0.52) in cognitively unimpaired participants. These changes in p-tau217 and pT217/T217 associated with CKD were again clearly lower than the fold increases in Aβ+ compared with Aβ− in patients with cognitive impairment (fold increase, 4.61 [95% CI, 3.39-5.83] and 4.11 [95% CI, 3.27-4.96]; *P* < .001) and cognitively unimpaired participants (fold increase, 1.27 [95% CI, 0.94-1.59] and 1.26 [95% CI, 0.98-1.55]; *P* < .001) (Figure 1). Correlations between plasma tau peptides and eGFR calculated using Lund-Malmö revised equation are shown in eTable 5 in Supplement 1. Associations of CKD with plasma Aβ42, Aβ40, and Aβ42/40 are presented in eTable 6 and eFigure 3 in Supplement 1.

## Discussion

Previous reports have suggested that CKD might influence plasma p-tau217 and p-tau181 concentrations determined using immunoassays.^2,16^ Here we corroborated these findings in cognitively unimpaired participants and 2 independent cohorts of patients with cognitive impairment using the IP-MS approach, which typically offers more accurate and reliable quantification of AD biomarkers in blood.^4,7^ We found that lower eGFR was associated with increased plasma levels of phosphorylated and unphosphorylated tau peptides that are measured simultaneously in the tau IP-MS assay. However, the correlations with eGFR were nonsignificant for pT217/T217 in patients with cognitive impairment in both cohorts as well as significantly attenuated for pT217/T217 in cognitively unimpaired participants and for pT181/T181 in both cognitively unimpaired participants and patients with cognitive impairment. Importantly, we demonstrate that there were no significant associations between CKD and the pT217/T217 ratio and changes in plasma pT181/T181 associated with CKD were small or nonsignificant. Our results indicate that by using p-tau/tau ratios, we may be able to reduce the variability in plasma p-tau levels driven by impaired kidney function and consequently such ratios are more robust measures of brain p-tau pathology in individuals with both early- and later-stage AD. This is likely true for the ratios of other related proteins, which is supported by the findings of attenuated associations of CKD with Aβ42/40 compared with Aβ42 and Aβ40 in the present study and in previous publications.^16,17^ Interestingly, we also found that the correlations of eGFR with p-tau217 (but not p-tau181) were weaker in patients with cognitive impairment than in cognitively unimpaired participants. Further, we report that in patients with cognitive impairment, Aβ positivity was associated with considerably larger increases in p-tau217 levels compared with the increases observed in CKD. Taken together, these results suggest that the effects CKD on p-tau217 levels might be diminishing in the presence of more advanced AD pathology. Of note, in agreement with prior data,^2^ the fold increases in p-tau217 and pT217/T217 were also clearly greater in Aβ+ cognitively unimpaired participants than in CKD+ cognitively unimpaired participants (albeit not as pronounced as in patients with cognitive impairment).^2^ The differences in the associations of CKD with p-tau217 and p-tau181 and with their respective ratios seen here could be related to the reported very low abundance of p-tau217 in the blood compared with p-tau181 in individuals without AD pathological changes together with the larger increase of p-tau217 in the central nervous system in response to AD pathology.^6,18^ In keeping, CKD appears to have a somewhat stronger impact on the definition of normal range and cutoffs for p-tau181 when compared with p-tau217.^2^

### Limitations

A relatively small number of participants with abnormal eGFR values prevented us from examining associations between plasma tau biomarkers and kidney dysfunction in CKD+ and CKD− groups separately, something that should be explored in future studies. Study participants were enrolled from the secondary (but not tertiary) specialized memory clinics and thus, investigations in more heterogeneous and ethnically and socioeconomically diverse population-based cohorts are also required. Even though we used 2 different equations to calculate eGFR with similar results, the findings of this study need confirmation with more updated methods to estimate glomerular filtration rate. Further, we used a single eGFR measure as an indicator of CKD, whereas CKD is clinically defined based on the presence of kidney damage or GFR less than 60 mL/min per 1.73m^2^ for 3 months or longer. However, it is highly unlikely that lower than normal eGFR in participants of the present study was due to the isolated acute increase in the creatinine levels because they were not collected at visits close to the emergency department visits.

## Conclusions

CKD was associated with a higher plasma concentration of soluble tau, but for p-tau217, the magnitude of this increase was much smaller than the increase associated with the presence of AD pathology. More importantly, there were no associations between CKD and the ratio of pT217/T217 and the associations with pT181/T181 were attenuated in patients with cognitive impairment. These novel findings suggest that plasma measures of the phosphorylated to unphosphorylated tau ratios are more accurate than p-tau forms alone as they correlate less with individual difference in glomerular filtration rate or impaired kidney function. Thus, to mitigate the effects of non-AD–related comorbidities like CKD on the performance of plasma AD biomarkers, certain tau ratios, and specifically pT217/T217, should be considered for implementation in clinical practice and drug trials.

## Notes

Supplement 1. eMethods. eTable 1. Demographic and clinical characteristics of patients with MCI, cohort-1 eTable 2. Demographic and clinical characteristics of the CKD- and CKD+ groups, cohort-1 eTable 3. Demographic and clinical characteristics of cognitively unimpaired and cognitively impaired participants, cohort-2 eTable 4. Demographic and clinical characteristics of the CKD- and CKD+ groups, cohort-2 eTable 5. Spearman correlations between plasma tau biomarkers and eGFR estimated using the Lund-Malmö-revised equation eTable 6. Spearman correlations between plasma Aβ biomarkers and eGFR estimated using the CKD-EPI equation and Lund-Malmö-revised equations eFigure 1. Associations between plasma tau biomarkers and estimated glomerular filtration rate (eGFR) in patients with mild cognitive impairment (MCI) in cohort-1 eFigure 2. Associations between plasma tau biomarkers and chronic kidney disease (CKD) estimated using the Lund-Malmö revised equation eFigure 3. Associations of plasma amyloid-β (Aβ) biomarkers with chronic kidney disease (CKD) and Aβ status eFigure 4. Associations between plasma tau biomarkers and estimated glomerular filtration rate (eGFR) in cognitively impaired (CI) participants in cohort-2 eFigure 5. Associations between plasma tau biomarkers and estimated glomerular filtration rate (eGFR) in cognitively unimpaired (CU) participants in cohort-2 eReferences

Supplement 2. Data sharing statement
