# Supplementary material for: Mitigating the Associations of Kidney Dysfunction With Blood Biomarkers of Alzheimer Disease by Using Phosphorylated Tau to Total Tau Ratios
Source: JAMA Neurol. 2023 Mar 29;80(5):516–22. doi: 10.1001/jamaneurol.2023.0199 (PMC10061310; doi:10.1001/jamaneurol.2023.0199)
Supplement: Supplement 1. — eMethods. eTable 1. Demographic and clinical characteristics of patients with MCI, cohort-1 eTable 2. Demographic and clinical characteristics of the CKD- and CKD+ groups, cohort-1 eTable 3. Demographic and clinical characteristics of cognitively unimpaired and cognitively impaired participants, cohort-2 eTable 4. Demographic and clinical characteristics of the CKD- and CKD+ groups, cohort-2 eTable 5. Spearman correlations between plasma tau biomarkers and eGFR estimated using the Lund-Malmö-revised equation eTable 6. Spearman correlations between plasma Aβ biomarkers and eGFR estimated using the CKD-EPI equation and Lund-Malmö-revised equations eFigure 1. Associations between plasma tau biomarkers and estimated glomerular filtration rate (eGFR) in patients with mild cognitive impairment (MCI) in cohort-1 eFigure 2. Associations between plasma tau biomarkers and chronic kidney disease (CKD) estimated using the Lund-Malmö revised equation eFigure 3. Associations of plasma amyloid-β (Aβ) biomarkers with chronic kidney disease (CKD) and Aβ status eFigure 4. Associations between plasma tau biomarkers and estimated glomerular filtration rate (eGFR) in cognitively impaired (CI) participants in cohort-2 eFigure 5. Associations between plasma tau biomarkers and estimated glomerular filtration rate (eGFR) in cognitively unimpaired (CU) participants in cohort-2 eReferences [file jamaneurol-e230199-s001.pdf]

Supplemental Online Content

Janelidze S, Barthélemy NR, He Y, Bateman RJ, Hansson O. Mitigating the associations of kidney dysfunction with blood biomarkers of Alzheimer disease by using phosphorylated tau to total tau ratios. *JAMA Neurol*. Published online March 29, 2023. doi:10.1001/jamaneurol.2023.0199

eMethods.
eTable 1. Demographic and clinical characteristics of patients with MCI, cohort-1
eTable 2. Demographic and clinical characteristics of the CKD- and CKD+ groups, cohort-1
eTable 3. Demographic and clinical characteristics of cognitively unimpaired and cognitively impaired participants, cohort-2
eTable 4. Demographic and clinical characteristics of the CKD- and CKD+ groups, cohort-2
eTable 5. Spearman correlations between plasma tau biomarkers and eGFR estimated using the Lund-Malmö-revised equation
eTable 6. Spearman correlations between plasma Aβ biomarkers and eGFR estimated using the CKD-EPI equation and Lund-Malmö-revised equations
eFigure 1. Associations between plasma tau biomarkers and estimated glomerular filtration rate (eGFR) in patients with mild cognitive impairment (MCI) in cohort-1
eFigure 2. Associations between plasma tau biomarkers and chronic kidney disease (CKD) estimated using the Lund-Malmö revised equation
eFigure 3. Associations of plasma amyloid-β (Aβ) biomarkers with chronic kidney disease (CKD) and Aβ status
eFigure 4. Associations between plasma tau biomarkers and estimated glomerular filtration rate (eGFR) in cognitively impaired (CI) participants in cohort-2
eFigure 5. Associations between plasma tau biomarkers and estimated glomerular filtration rate (eGFR) in cognitively unimpaired (CU) participants in cohort-2
eReferences.

This supplemental material has been provided by the authors to give readers additional information about their work.

© 2023 Janelidze S et al. *JAMA Neurology.*

eMethods

Participants

The study included 2 cohorts, cohort-1 and a validation cohort-2 with participants recruited at the Memory Clinic at Skåne University Hospital in Malmö, Sweden, between 2000 and 2005 in cohort-1 and between 2017 and 2022 in cohort-2. Cohort-1 included 141 individuals with a clinical diagnosis of mild cognitive impairment (MCI) who all underwent a thorough physical, neurological, and psychiatric examination, as well as a clinical interview focusing on cognitive symptoms and activities of daily living function by physicians with an expertise in cognitive disorders.[1, 2] Patients with MCI had to fulfill the criteria by Petersen,[3] including (1) memory complaint, preferably corroborated by an informant; (2) objective memory impairment adjusted for age and education, as judged by the physician; (3) preservation of general cognitive functioning, as determined by the clinician's judgment based on a structured interview with the patient and a Mini Mental Status Examination (MMSE) score greater than or equal to 24; (4) zero or minimal impairment of daily life activities; and (5) not fulfilling the DSM-IIIR criteria for dementia. The exclusion criteria were (1) significant unstable systemic illness or organ failure; (2) current significant alcohol or substance misuse; and (3) cognitive impairment that could be explained by other specific non-neurodegenerative disorders such as brain tumor or subdural hematoma.

Cohort-2 included a group of cognitively unimpaired people (CU)[4] comprising 104 cognitively healthy controls and 56 individuals with subjective cognitive decline (SCD) and another cognitively impaired (CI) group of 172 patients with MCI (N=98) or dementia (N=74) from the BioFINDER-2 study[5] who had plasma tau assessments using IP-MS-based method developed at Washington University. The inclusion criteria for controls were i) ages 40-100 years; ii) absence of cognitive symptoms as assessed by a physician specialized in cognitive disorders; iii) MMSE score of 27-30 points (40-65 years of age) or 26-30 points (66-100 years of age) at screening visit; iv) do not fulfill the criteria for mild or major neurocognitive disorder (MCI or dementia) according to DSM-5[6]; and v) fluent in Swedish. The recruitment process of neurologically and cognitively healthy controls was designed to build two study populations with 50% APOE *ε4* carriers in each. Inclusion criteria for patients with SCD or MCI were: i) age 40-100 years; ii) referred to the memory clinics due to cognitive symptoms; iii) MMSE score of 24-30 points; iv) does not fulfill the criteria for any dementia (major neurocognitive disorder) according to DSM-5, v) fluency in Swedish. Participants were classified as having MCI if they performed worse than -1.5 SD in any cognitive domain according to test norms adjusted for age and education. The neuropsychological battery covered the domains attention/executive function (Trail Making Test A and B and Symbol Digit Modalities Test), verbal ability (verbal fluency animals and the 15-word short version of the Boston Naming Test), memory (10-word delayed recall from the Alzheimer's Disease (AD) Assessment Scale [ADAS]), and visuospatial function (incomplete letters and cube analysis from the Visual Object and Space Perception battery [VOSP]). Patients that were not classified as MCI were considered to have SCD. Dementia group included individuals with AD dementia (ADD, N=56), vascular dementia (VaD, N=5), dementia with Lewy bodies (DLB, N=3), progressive supranuclear palsy (PSP, N=2), behavioral variant frontotemporal dementia (bvFTD, N=3), semantic variant primary progressive aphasia (svPPA, N=1) and dementia not otherwise specified (N=4). Inclusion criteria for ADD were: i) ages 40-100 years; ii) referred to the memory clinics due to cognitive symptoms; iii) MMSE score of ≥12 points; iv) fulfill the DSM-5 criteria for dementia (major neurocognitive disorder) due to AD[6]; and v) fluent in Swedish. Only participants who were 50 years of age or older were included in this study. Clinical ADD was diagnosed according to the DSM-5 criteria for major neurocognitive disorder due to AD[6] and the requirement that they were Aβ-positive in agreement with the updated NIA-AA criteria for AD[4]. Inclusion criteria for non-ADD were: i) ages 40-100 years; ii) fulfillment of criteria for dementia (major neurocognitive disorder) due to DLB, FTD, DLB, VaD[6], PSP[7], or svPPA[8]; and iii) fluent in Swedish. Exclusion criteria in cohort-2 were: i) significant unstable systemic illness that makes it difficult to participate in the study; ii) current significant alcohol or substance misuse; iii) refusing lumbar puncture, MRI or PET.

Plasma and CSF sampling

CSF and blood samples were drawn in the morning while participants were not necessarily non-fasting. Blood was collected in six K2-EDTA-plasma tubes and centrifuged at 2000g, +4°C for 10 minutes. Following centrifugation plasma was aliquoted into 1.5-ml polypropylene tubes (1 ml per tube) and stored at −80°C. CSF was obtained by lumbar puncture and stored at −80°C in polypropylene tubes following the Alzheimer's Association flow chart for lumbar puncture and CSF sample processing.[9] All samples went through one freeze-thaw cycle before the analysis when 0.2-0.5ml were further aliquoted into LoBind tubes. Plasma levels of p-tau217, p-tau181, Tau212-221, Tau181-190 and pT217/T217 and pT181/T181 ratios were measured using an in-house multiplex immunoprecipitation-mass spectrometry (IP-MS) assay at the Department of Neurology, Washington University School of Medicine (St. Louis, MO, USA) as previously described[10] with some modifications. Briefly, immuno-purification was performed as the first purification step using tau antibody immobilized on magnetic beads instead of sepharose beads. Two steps of solid phase extraction were performed at the protein then the peptide level, respectively. One ml of plasma was mixed with ^{15}N-labeled tau then immuno-precipitated using Tau1 antibody (anti tau-192-199) cross-linked to Dynabeads M270 Epoxy magnetic beads. Tau precipitate was eluted and extracted on HLB µ-elution plate. After drying the eluate,

© 2023 Janelidze S et al. *JAMA Neurology.*

mixture of labeled synthetic peptides for each analyte was added and the sample was digested with trypsin. The digest was extracted on HLB μ-elution plate; eluate was dried then resuspended prior to nanoLC-MS/HRMS analysis. Plasma concentrations of Aβ40 and Aβ42 were determined with immunoassays (ADx Neurosciences) in cohort-1 and antibody-free liquid chromatography-MS (Araclon Biotech)[11] in cohort-2. CSF Aβ40 and Aβ42 concentrations were assessed using immunoassays (Meso Scale Discovery) in cohort-1 and using the Elecsys (Roche Diagnostics) or Lumipulse G (Fujirebio) immunoassays in cohort-2. All samples were analyzed by staff blinded to the clinical data.

Tau-PET image acquisition and processing

PET imaging was performed on a digital GE Discovery MI scanner as previously described.[5] Scans were acquired 70–90 min post injection of ~370 MBq [^{18}F]RO948 and images were processed according to the pipeline described previously.[12] Briefly, PET images were attenuation corrected, motion corrected, summed and registered to the closest T1-weighted MRI processed through the longitudinal pipeline of FreeSurfer version 6.0. Standardized uptake value ratio (SUVR) images were created using the inferior cerebellar gray matter as the reference region. [^{18}F]RO94858 SUVRs were obtained for a temporal meta-ROI composed of entorhinal cortex, inferior and middle temporal cortices, fusiform gyrus, parahippocampal cortex and amygdala that corresponding to Braak I-IV regions.[13]

© 2023 Janelidze S et al. *JAMA Neurology.*

eTable 1. Demographic and clinical characteristics of patients with MCI, cohort-1

	**Overall**	**Aβ-**[a]	**Aβ+**[a]
N	141	74	67
Age, years	73.0 (65.0-78.5)	70.5 (63.0-77.3)	75.0 (70.0-79.0)
Female, n (%)	82 (58.2)	40 (54.1)	42 (62.7)
MMSE	27.0 (26.0-29.0)	28.0 (27.0-29.0)	27.0 (25.0-28.0)
APOE ε4 positivity, n (%)	79 (56.0)	26 (35.1)	53 (79.1)
eGFR	72.6 (60.5-82.3)	72.0 (61.2-83.9)	72.6 (59.0-81.6)
Plasma tau			
p-tau217, pg/ml	3.61 (2.07-8.91)	2.14 (1.69-3.29)	8.78 (5.35-13.05)
Tau212-221, pg/ml	276.8 (238.4-334.3)	270.1 (236.6-314.0)	282.5 (241.9-347.2)
pT217/T217, %	1.28 (0.74-3.12)	0.770 (0.654-1.049)	3.07 (1.93-4.40)
p-tau181, pg/ml	47.2 (37.4-64.6)	40.8 (32.8-53.2)	58.4 (45.7-80.3)
Tau181-190, pg/ml	203.3 (173.5-262.5)	191.9 (166.5-249.9)	210.4 (179.4-273.0)
pT181/T181, %	23.0 (19.5-28.3)	20.3 (18.3-22.9)	27.9 (23.7-31.1)
Plasma Aβ			
Aβ42	15.8 (14.1-18.2)	16.7 (14.1-18.9)	15.3 (14.1-17.6)
Aβ40	136.9 (124.3-156.3)	131.6 (123.4-157.0)	143.1 (124.6-155.3)
Aβ42/40	0.115 (0.105-0.125)	0.121 (0.108-0.131)	0.112 (0.104-0.119)

Data are shown as median (interquartile range) unless otherwise specified.
[a] Aβ status was defined using CSF Aβ42/40 binarized based on the previously described threshold of 0.07.[2, 14]

Abbreviations: Aβ, amyloid-β; eGFR, estimated glomerular filtration rate; MCI, mild cognitive impairment; MMSE, Mini Mental State Examination; p-tau, phosphorylated tau.

© 2023 Janelidze S et al. *JAMA Neurology.*

eTable 2. Demographic and clinical characteristics of the CKD- and CKD+ groups, cohort-1

	**Overall**	**CKD-**[a]	**CKD+** [a]
N	141	106	35
Age, years	73.0 (65.0-78.5)	72.0 (65.0-77.8)	77.0 (71.5-80.5)
Female, n (%)	82 (58.2)	47 (44.3)	12 (34.3)
MMSE	27.0 (26.0-29.0)	28.0 (26.0-29.0)	27.0 (25.5-28.5)
APOE ε4 positivity, n (%)	79 (56.0)	45 (42.5)	17 (48.6)
Plasma tau			
p-tau217, pg/ml	3.61 (2.07-8.91)	3.32 (1.99-7.35)	5.59 (3.29-11.24)
Tau212-221, pg/ml	276.8 (238.4-334.3)	260.3 (226.3-307.4)	340.2 (277.9-383.7)
pT217/T217, %	1.28 (0.74-3.12)	1.23 (0.72-2.90)	1.84 (0.89-3.42)
p-tau181, pg/ml	47.2 (37.4-64.6)	43.6 (34.7-55.6)	67.0 (54.8-86.2)
Tau181-190, pg/ml	203.3 (173.5-262.5)	191.4 (166.9-228.1)	279.3 (223.5-309.4)
pT181/T181, %	23.0 (19.5-28.3)	22.0 (19.2-27.4)	26.2 (22.4-30.2)
Plasma Aβ			
Aβ42	15.8 (14.1-18.2)	15.3 (13.4-17.3)	18.7 (15.7-20.6)
Aβ40	136.9 (124.3-156.3)	130.7 (122.6-146.5)	163.7 (144.7-171.7)
Aβ42/40	0.115 (0.105-0.125)	0.114 (0.105-0.125)	0.117 (0.109-0.124)

Data are shown as median (interquartile range) unless otherwise specified.
[a] CKD status was defined based on the threshold of <60 ml/min/1.73m2 as described in the methods.[15]

Abbreviations: Aβ, amyloid-β; CKD, chronic kidney disease; MMSE, Mini Mental State Examination; p-tau, phosphorylated tau.

© 2023 Janelidze S et al. *JAMA Neurology.*

eTable 3. Demographic and clinical characteristics of cognitively unimpaired and cognitively impaired participants, cohort-2

	**Overall**	**CU**	**CI**
N	332	160	172
Age, years	72.0 (61.6 - 77.1)	65.7 (58.0 - 76.1)	74.0 (68.5 - 77.7)
Female, n (%)	169 (50.9)	88 (55.0)	81 (47.1)
MMSE [a]	28.0 (25.0 - 29.0)	29.0 (28.0 - 30.0)	25.0 (21.8 - 28.0)
APOE ε4 positivity, n (%) [b]	162 (48.8)	69 (43.1)	93 (54.1)
Aβ positivity, n (%) [c]	172 (51.8)	50 (31.3)	122 (70.9)
eGFR [d]	81.7 (70.8 - 88.5)	81.4 (72.4 - 88.7)	81.8 (69.0 - 88.4)
Tau-PET SUVR [e]	1.20 (1.12 - 1.38)	1.14 (1.08 - 1.21)	1.30 (1.17 - 1.79)
Plasma tau			
p-tau217, pg/ml	1.28 (0.74 - 3.25)	0.874 (0.641 - 1.29)	2.63 (1.09 - 4.96)
Tau212-221, pg/ml	138.6 (117.2 - 167.9)	135.6 (117.1 - 161.6)	142.3 (117.2 - 176.8)
pT217/T217, %	0.862 (0.555 - 2.171)	0.628 (0.511 - 0.883)	1.924 (0.710 - 3.152)
p-tau181, pg/ml	32.9 (23.5 - 45.2)	29.2 (21.8 - 37.0)	39.1 (26.6 - 51.6)
Tau181-190, pg/ml	97.8 (81.5 - 123.0)	94.9 (79.6 - 114.2)	101.8 (83.3 - 130.0)
pT181/T181, %	32.6 (27.6 - 38.5)	29.9 (27.0 - 34.4)	36.7 (29.6 - 42.9)
Plasma Aβ [f]			
Aβ42	62.2 (53.2 - 69.6)	64.2 (56.1 - 73.9)	57.3 (51.8 - 67.1)
Aβ40	300.7 (266.9 - 332.0)	303.7 (266.5 - 331.2)	300.1 (267.6 - 334.2)
Aβ42/40	0.208 (0.186 - 0.233)	0.217 (0.197 - 0.241)	0.202 (0.174 - 0.221)

Data are shown as median (interquartile range) unless otherwise specified.
[a] MMSE was missing for 6 participants.
[b] *APOE* ε4 was missing for 26 participants.
[c] Aβ status was missing for 1 participant; Aβ status was defined using CSF Aβ42/40 binarized as described in the methods.
[d] eGFR was estimated using the CKD-EPI equation.
[e] Tau-PET data was available in 301 participants.
[f] Plasma Aβ data was available in 212 participants.

Abbreviations: Aβ, amyloid-β; CI, cognitively impaired; CU, cognitively unimpaired; eGFR, estimated glomerular filtration rate; MMSE, Mini Mental State Examination; p-tau, phosphorylated tau; PET, positron emission tomography; SUVR, standardized uptake value ratio.

© 2023 Janelidze S et al. *JAMA Neurology.*

eTable 4. Demographic and clinical characteristics of the CKD- and CKD+ groups, cohort-2

	**Overall**	**CKD-[a]**	**CKD+[a]**
N	332	295	37
Age, years	72.0 (61.6 - 77.1)	71.2 (61.1 - 76.6)	76.4 (70.2 - 80.8)
Female, n (%)	169 (50.9)	146 (49.5)	17 (45.9)
MMSE[b]	28.0 (25.0 - 29.0)	28.0 (25.0 - 29.0)	28.0 (24.0 - 29.0)
APOE ε4 positivity, n (%)[c]	162 (48.8)	143 (48.5)	19 (51.4)
Tau-PET SUVR[d]	1.20 (1.12 - 1.38)	1.21 (1.12 - 1.40)	1.16 (1.09 - 1.22)
Plasma tau			
p-tau217, pg/ml	1.28 (0.74 - 3.25)	1.13 (0.69 - 2.91)	2.64 (1.46 - 4.80)
Tau212-221, pg/ml	138.6 (117.2 - 167.9)	133.8 (114.7 - 157.4)	199.6 (170.7 - 243.0)
pT217/T217, %	0.862 (0.555 - 2.171)	0.814 (0.539 - 2.157)	1.21 (0.751 - 2.25)
p-tau181, pg/ml	32.9 (23.5 - 45.2)	30.9 (22.4 - 41.2)	53.2 (41.1 - 69.5)
Tau181-190, pg/ml	97.8 (81.5 - 123.0)	95.0 (79.3 - 115.5)	144.9 (124.8 - 180.8)
pT181/T181, %	32.6 (27.6 - 38.5)	32.3 (27.5 - 38.2)	35.9 (29.8 - 39.1)
Plasma Aβ[e]			
Aβ42	62.2 (53.2 - 69.6)	60.3 (52.7 - 68.4)	79.5 (61.8 - 94.4)
Aβ40	300.7 (266.9 - 332.0)	294.7 (265.0 - 325.2)	354.6 (315.3 - 437.9)
Aβ42/40	0.208 (0.186 - 0.233)	0.208 (0.184 - 0.232)	0.219 (0.194 - 0.241)

Data are shown as median (interquartile range) unless otherwise specified.
[a] CKD status was defined based on the threshold of <60 ml/min/1.73m2 as described in the methods.[15]
[b] MMSE was missing for 6 participants
[c] *APOE* ε4 was missing for 26 participants
[d] Tau-PET data was available in 301 participants
[e] Plasma Aβ data was available in 212 participants

Abbreviations: Aβ, amyloid-β; CKD, chronic kidney disease; MMSE, Mini Mental State Examination; p-tau, phosphorylated tau; PET, positron emission tomography; SUVR, standardized uptake value ratio.

© 2023 Janelidze S et al. *JAMA Neurology.*

eTable 5. Spearman correlations between plasma tau biomarkers and eGFR estimated using the Lund-Malmö-revised equation

	R (95% CI, p-value)	*R_{diff} vs pT217/T217 (95% CI, p-value)*		*R (95% CI, p-value)*	*R_{diff} vs pT181/T181 (95% CI, p-value)*
**Cohort 1, MCI**					
**p-tau217**	-0.29 (-0.45 to -0.12, p<0.001)	-0.12 (-0.17 to -0.07, p<0.001)	**p-tau181**	-0.54 (-0.65 to -0.40, p<0.001)	-0.25 (-0.34 to -0.16, p<0.001)
**Tau212-221**	-0.50 (-0.61 to -0.36, p<0.001)	-0.32 (-0.51 to -0.13, p=0.001)	**Tau181-190**	-0.59 (-0.70 to -0.46, p<0.001)	-0.30 (-0.44 to -0.16, p<0.001)
**pT217/T217**	-0.18 (-0.34 to -0.01, p=0.037)	NA	**pT181/T181**	-0.29 (-0.43 to -0.13, p<0.001)	NA
**Cohort 2, CU**					
**p-tau217**	-0.52 (-0.63 to -0.39, p<0.001)	-0.14 (-0.21 to -0.06, p=0.001)	**p-tau181**	-0.53 (-0.63 to -0.40, p=8.9E-13)	-0.31 (-0.43 to -0.19, p<0.001)
**Tau212-221**	-0.44 (-0.57 to -0.30, p<0.001)	-0.06 (-0.25 to 0.14, p=0.57)	**Tau181-190**	-0.53 (-0.64 to -0.40, p=6.7E-13)	-0.31 (-0.49 to -0.13, p<0.001)
**pT217/T217**	-0.38 (-0.51 to -0.25, p<0.001)	NA	**pT181/T181**	-0.22 (-0.36 to -0.07, p=0.006)	NA
**Cohort 2, CI**					
**p-tau217**	-0.17 (-0.31 to -0.03, p=0.024)	-0.15 (-0.20 to -0.10, p<0.001)	**p-tau181**	-0.39 (-0.52 to -0.24, p<0.001)	-0.33 (-0.45 to -0.22, p<0.001)
**Tau212-221**	-0.51 (-0.62 to -0.39, p<0.001)	-0.49 (-0.66 to -0.32, p<0.001)	**Tau181-190**	-0.53 (-0.64 to -0.41, p<0.001)	-0.47 (-0.64 to -0.31, p<0.001)
**pT217/T217**	-0.02 (-0.17 to 0.13, p=0.79)	NA	**pT181/T181**	-0.06 (-0.21 to 0.09, p=0.46)	NA

Data are shown as Spearman correlation coefficients (Rho), 95% CI and p-value.
Abbreviations: CI, confidence interval; eGFR, estimated glomerular filtration rate; NA, not applicable; p-tau, phosphorylated tau

© 2023 Janelidze S et al. *JAMA Neurology.*

eTable 6. Spearman correlations between plasma Aβ biomarkers and eGFR estimated using the CKD-EPI equation and Lund-Malmö-revised equations

	**eGFR (CKD-EPI equation)**		**eGFR (Lund-Malmö-Revised equation)**	
	R (95% CI, p-value)	*R_{diff} vs Aβ42/Aβ40 (95% CI, p-value)*	*R (95% CI, p-value)*	*R_{diff} vs Aβ42/Aβ40 (95% CI, p-value)*
**Cohort 1, MCI**				
Aβ42	-0.46 (-0.61 to -0.30, p<0.001)	-0.41 (-0.56 to -0.27, p<0.001)	-0.46 (-0.60 to -0.30, p<0.001)	-0.45 (-0.59 to -0.32, p<0.001)
Aβ40	-0.55 -0.66 to -0.41, p<0.001)	-0.50 (-0.70 to -0.30, p<0.001)	-0.58 (-0.70 to -0.45, p<0.001)	-0.57 (-0.76 to -0.39, p<0.001)
Aβ42/Aβ40	-0.05 (-0.21 to 0.12, p=0.58)	NA	-0.01 (-0.18 to 0.16, p=0.93)	NA
**Cohort 2, CU [a]**				
Aβ42	-0.37 (-0.55 to -0.19, p<0.001)	-0.36 (-0.68 to -0.06, p=0.018)	-0.38 (-0.55 to -0.20, p<0.001)	-0.37 (-0.68 to -0.06, p=0.018)
Aβ40	-0.39 (-0.55 to -0.21, p<0.001)	-0.38 (-0.63 to -0.15, p=0.002)	-0.41 (-0.57 to -0.23, p<0.001)	-0.40 (-0.63 to -0.18, p<0.001)
Aβ42/Aβ40	0.005 (-0.19 to 0.18, p=0.95)	NA	-0.008 (-0.19 to 0.18, p=0.93)	NA
**Cohort 2, CI [a]**				
Aβ42	-0.44 (-0.60 to -0.26, p<0.001)	-0.39 (-0.57 to -0.22, p=0.0005)	-0.46 (-0.61 to -0.28, p<0.001)	-0.42 (-0.59 to -0.25, p<0.001)
Aβ40	-0.54 (-0.67 to -0.38, p<0.001)	-0.49 (-0.78 to -0.22, p=0.001)	-0.56 (-0.70 to -0.40, p<0.001)	-0.52 (-0.80 to -0.25, p=0.001)
Aβ42/Aβ40	-0.050 (-0.25 to 0.15, p=0.63)	NA	-0.042 (-0.26 to 0.16, p=0.68)	NA

Data are shown as Spearman correlation coefficients (Rho), 95% CI and p-value.
[a] In cohort-2, plasma Aβ data were available in 113 CU and 99 CU.

Abbreviations: Aβ, amyloid-β; CI, confidence interval; eGFR, estimated glomerular filtration rate; NA, not applicable.

© 2023 Janelidze S et al. *JAMA Neurology.*

eFigure 1

MCI, cohort-1

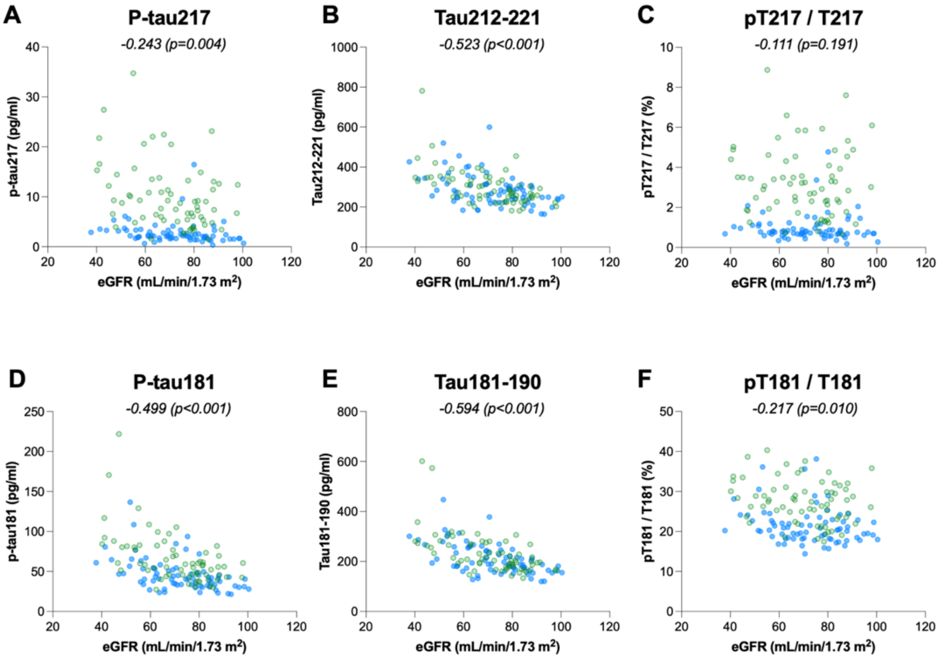

Associations between plasma tau biomarkers and estimated glomerular filtration rate (eGFR) in patients with mild cognitive impairment (MCI) in cohort-1. Associations of eGFR with plasma p-tau217 (**A**), Tau212-221 (**B**), pT217/T217 (**C**), p-tau181 (**D**), Tau181-190 (**E**) and pT181/T181 (**F**). Data are shown Spearman Rho (p value). Blue and green circles indicate CSF Aβ- and Aβ+, respectively. eGFR was estimated using the CKD-EPI equation.

eFigure 2

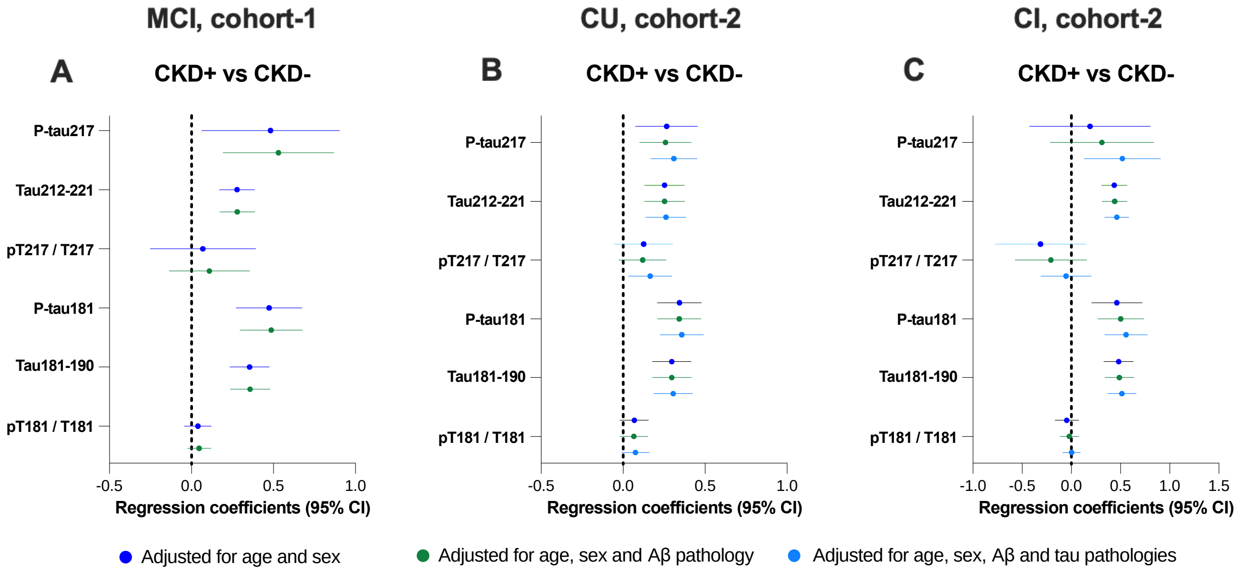

Associations between plasma tau biomarkers and chronic kidney disease (CKD) estimated using the Lund-Malmö revised equation. Fold increase in plasma biomarker levels in participants with CKD (CKD+) compared with those without CKD (CKD-) in cohort-1 (MCI, N=141; **A**) and cohort-2 (CU, N=146; **B** and CI, N=154; **C**). Data are coefficients with 95% CI from linear regression models.

Abbreviations: CI, cognitively impaired; CU, cognitively unimpaired; MCI, mild cognitive impairment

© 2023 Janelidze S et al. *JAMA Neurology.*

eFigure 3

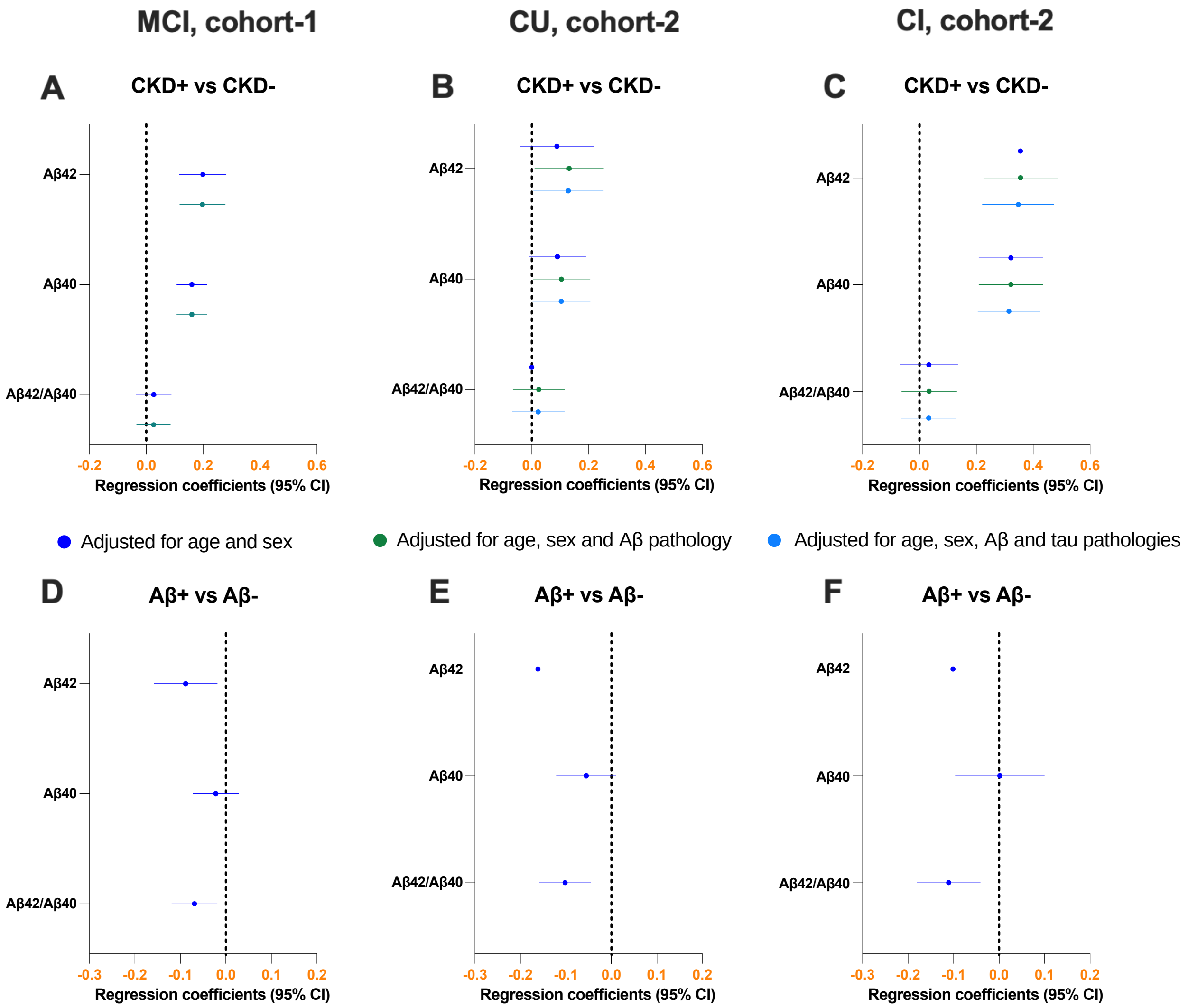

Associations of plasma amyloid-β (Aβ) biomarkers with chronic kidney disease (CKD) and Aβ status. Fold increase in plasma biomarker levels in participants with CKD (CKD+) compared with those without CKD (CKD-) in cohort-1 (MCI, N=141; **A**) and cohort-2 (CU, N=109; **B** and CI, N=95; **C**) and in Aβ+ participants compared with Aβ− participants in cohort-1 (MCI, N=141; **D**) and cohort-2 (CU, N=109; **E** and CI**,** N=95; **F**). Data are coefficients with 95% CI from linear regression models. Aβ status was defined using CSF Aβ42/40.
Abbreviations: Aβ, amyloid-β; CI, cognitively impaired; CU, cognitively unimpaired; CSF, cerebrospinal fluid; MCI, mild cognitive impairment

© 2023 Janelidze S et al. *JAMA Neurology.*

eFigure 4

CI, cohort-2

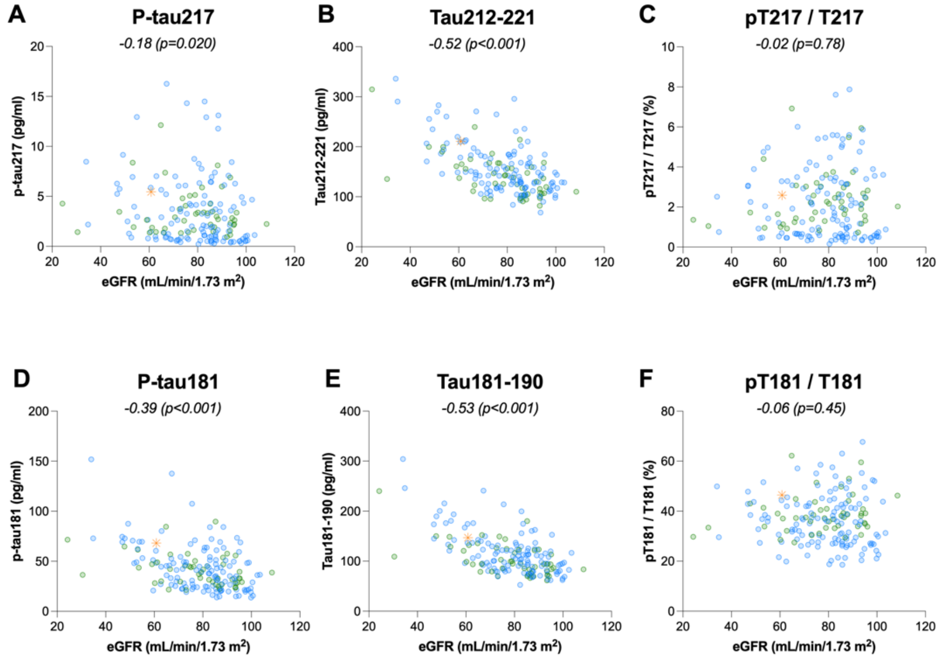

Associations between plasma tau biomarkers and estimated glomerular filtration rate (eGFR) in cognitively impaired (CI) participants in cohort-2. Associations of GFR with plasma p-tau217 (**A**), Tau212-221 (**B**), pT217/T217 (**C**), p-tau181 (**D**), Tau181-190 (**E**) and pT181/T181 (**F**). Data are shown Spearman Rho (p value). Blue and green circles indicate CSF Aβ- and Aβ+, respectively; CSF Aβ data were missing for 1 participant with AD dementia indicated in asterisk. eGFR was estimated using the CKD-EPI equation.

eFigure 5

CU, cohort-2

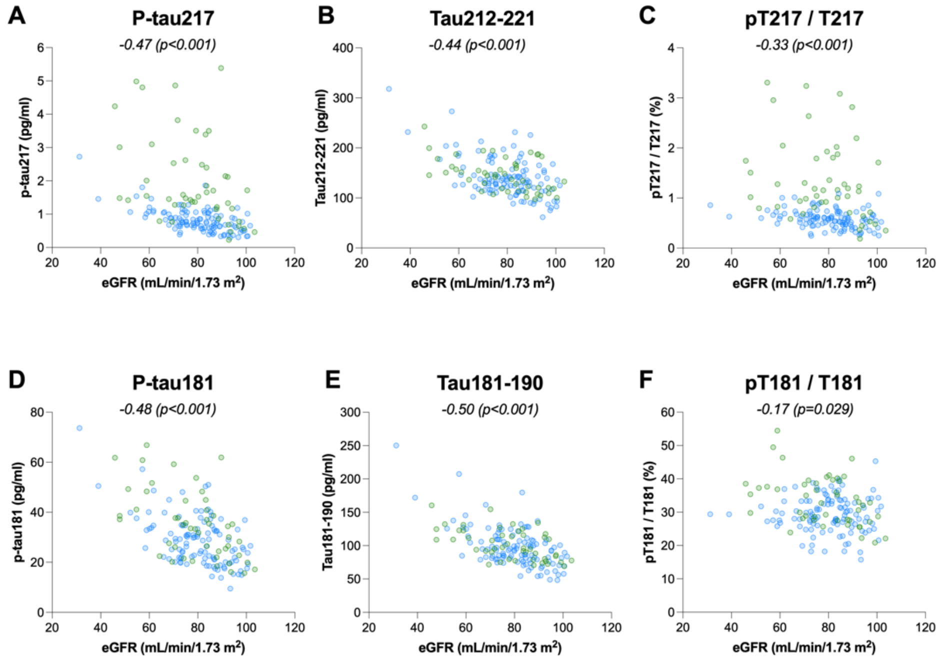

Associations between plasma tau biomarkers and estimated glomerular filtration rate (eGFR) in cognitively unimpaired (CU) participants in cohort-2. Associations of GFR with plasma p-tau217 (**A**), Tau212-221 (**B**), pT217/T217 (**C**), p-tau181 (**D**), Tau181-190 (**E**) and pT181/T181 (**F**). Data are shown Spearman Rho (p value). Blue and green circles indicate CSF Aβ- and Aβ+, respectively. eGFR was estimated using the CKD-EPI equation.

© 2023 Janelidze S et al. *JAMA Neurology.*

eReferences

1. Hertze J, Minthon L, Zetterberg H, Vanmechelen E, Blennow K, Hansson O. Evaluation of CSF biomarkers as predictors of Alzheimer's disease: a clinical follow-up study of 4.7 years. *J Alzheimers Dis*. 2010;21(4):1119-28. doi:10.3233/jad-2010-100207
2. Janelidze S, Bali D, Ashton NJ, et al. Head-to-head comparison of 10 plasma phospho-tau assays in prodromal Alzheimer's disease. *Brain*. 2022;doi:10.1093/brain/awac333
3. Petersen RC. Mild cognitive impairment as a diagnostic entity. *J Intern Med*. 2004;256(3):183-94. doi:10.1111/j.1365-2796.2004.01388.x
4. Jack CR, Jr., Bennett DA, Blennow K, et al. NIA-AA Research Framework: Toward a biological definition of Alzheimer's disease. *Alzheimers Dement*. 2018;14(4):535-562. doi:10.1016/j.jalz.2018.02.018
5. Palmqvist S, Janelidze S, Quiroz YT, et al. Discriminative Accuracy of Plasma Phospho-tau217 for Alzheimer Disease vs Other Neurodegenerative Disorders. *JAMA*. 2020;doi:10.1001/jama.2020.12134
6. Association AP. *Diagnostic and Statistical Manual of Mental Disorders*. 5th Edition ed.
7. Hoglinger GU, Respondek G, Stamelou M, et al. Clinical diagnosis of progressive supranuclear palsy: The movement disorder society criteria. *Mov Disord*. 2017;32(6):853-864. doi:10.1002/mds.26987
8. Gorno-Tempini ML, Hillis AE, Weintraub S, et al. Classification of primary progressive aphasia and its variants. *Neurology*. 2011;76(11):1006-14. doi:10.1212/WNL.0b013e31821103e6
9. Blennow K, Hampel H, Weiner M, Zetterberg H. Cerebrospinal fluid and plasma biomarkers in Alzheimer disease. *Nat Rev Neurol*. 2010;6(3):131-44. doi:10.1038/nrneurol.2010.4
10. Barthelemy NR, Horie K, Sato C, Bateman RJ. Blood plasma phosphorylated-tau isoforms track CNS change in Alzheimer's disease. *J Exp Med*. 2020;217(11)doi:10.1084/jem.20200861
11. Janelidze S, Teunissen CE, Zetterberg H, et al. Head-to-Head Comparison of 8 Plasma Amyloid-beta 42/40 Assays in Alzheimer Disease. *JAMA Neurol*. 2021;78(11):1375-1382. doi:10.1001/jamaneurol.2021.3180
12. Leuzy A, Smith R, Ossenkoppele R, et al. Diagnostic Performance of RO948 F 18 Tau Positron Emission Tomography in the Differentiation of Alzheimer Disease From Other Neurodegenerative Disorders. *JAMA Neurol*. 2020;doi:10.1001/jamaneurol.2020.0989
13. Cho H, Choi JY, Hwang MS, et al. In vivo cortical spreading pattern of tau and amyloid in the Alzheimer disease spectrum. *Ann Neurol*. 2016;80(2):247-58. doi:10.1002/ana.24711
14. Groot C, Cicognola C, Bali D, et al. Diagnostic and prognostic performance to detect Alzheimer's disease and clinical progression of a novel assay for plasma p-tau217. *Alzheimers Res Ther*. 2022;14(1):67. doi:10.1186/s13195-022-01005-8
15. Levey AS, de Jong PE, Coresh J, et al. The definition, classification, and prognosis of chronic kidney disease: a KDIGO Controversies Conference report. *Kidney Int*. 2011;80(1):17-28. doi:10.1038/ki.2010.483

© 2023 Janelidze S et al. *JAMA Neurology.*
