# Supplementary material for: Mitigating the Associations of Kidney Dysfunction With Blood Biomarkers of Alzheimer Disease by Using Phosphorylated Tau to Total Tau Ratios
Source: JAMA Neurol. 2023 Mar 29;80(5):516–22. doi: 10.1001/jamaneurol.2023.0199 (PMC10061310; doi:10.1001/jamaneurol.2023.0199)
Supplement: Supplement 2. — Data sharing statement [file jamaneurol-e230199-s002.pdf]

Data Sharing Statement

Janelidze. Mitigating the Associations of Kidney Dysfunction with Blood Biomarkers of Alzheimer Disease by Using Phosphorylated Tau to Total Tau Ratios. *JAMA Neurol*. Published March 29, 2023. doi:10.1001/jamaneurol.2023.0199

Data
Data available: Yes
Data types: Other (please specify)
Additional Information: Anonymized data will be shared by request from a qualified academic investigator for the sole purpose of replicating procedures and results presented in the article and as long as data transfer is in agreement with EU legislation on the general data protection regulation and decisions by the Ethical Review Board of Sweden and Region Skåne, which should be regulated in a material transfer agreement.
How to access data: As above.
When available: With publication

Supporting Documents
Document types: None
